# Generative enhancement of suboptimally opacified cerebral CT angiography for large-vessel occlusion detection: a multireader study

**DOI:** 10.3389/fneur.2026.1955596

**Published:** 2026-10-13

**Authors:** Yifan Tu, Mengfei Wang, Zheng Sun, Yao Zeng, Yunyi Li, Yixiao Tang, Yulu Yang, Zhongzheng Cao, Shundong Hu, Dan Wang, Yuehua Li

**Affiliations:** 1 Department of Radiology, Shanghai Sixth People's Hospital Affiliated to Shanghai Jiao Tong University School of Medicine, Shanghai, China; 2 Donghua University, Shanghai, China; 3 Faculty of Medical Imaging Technology, College of Health Science and Technology, Shanghai Jiao Tong University School of Medicine, Shanghai, China

**Keywords:** acute ischemic stroke, CT angiography, deep learning, generative adversarial network, image synthesis, large-vessel occlusion, multireader multicase study

## Abstract

**Background:**

Rapid identification of proximal anterior circulation large-vessel occlusion (LVO) on CT angiography (CTA) is essential for thrombectomy triage. Suboptimal arterial phase timing can obscure the intracranial arteries, prompting repeat CTA, additional contrast exposure, and treatment delay. We evaluated whether deep-learning-generated vascular images from a single suboptimally opacified acquisition improved LVO detection.

**Methods:**

This retrospective study included 626 patients. RescueCTA, a paired 2.5D conditional generative model, was developed in 391 patients using last-frame CT perfusion (CTP) as the simulated acquisition-failure input and same-session CTA as the target. Evaluation included 194 primary cases with simulated inputs and 41 exploratory cases with genuinely underopacified CTA. RescueCTA was compared with three generative baselines. Two radiologists assessed image quality. Four readers compared source images alone with source images plus RescueCTA for target LVO detection in the primary cohort; two also evaluated the exploratory cohort. The primary endpoint was the multireader multicase (MRMC) area under the receiver operating characteristic curve (AUC); exploratory analyses were descriptive.

**Results:**

In the primary cohort, RescueCTA image quality was higher than that of all three baselines (3.90 ± 0.81). Combined reading improved AUC from 0.850 to 0.933 (ΔAUC = 0.083 [95% confidence interval (CI), 0.025–0.141]; *p* = 0.007), while paired CTA reached 0.948 (*p* = 0.594 vs. combined reading). Strict AUC improved from 0.756 to 0.880 (*p* < 0.001) but remained lower than that of paired CTA (0.935; *p* = 0.019). In the exploratory cohort, combined reading yielded a higher AUC than underopacified CTA alone (0.981 vs. 0.889).

**Conclusion:**

Deep-learning-generated vascular images improved LVO detection under simulated underopacification and showed preliminary feasibility in genuinely underopacified CTA. Larger real-world cohorts are needed to establish diagnostic performance after suboptimal arterial phase CTA.

## 1 Introduction

Stroke is one of the leading causes of death and long-term disability worldwide (1, 2), and the timely identification of candidates for reperfusion therapy is critical to improving outcomes (3–5). Emergency CT angiography (CTA) enables rapid detection and localization of proximal anterior circulation large-vessel occlusion (LVO) and is an important imaging basis for mechanical thrombectomy decisions (6–8). Its arterial opacification, however, depends heavily on acquisition timing. Interindividual variation in circulation time, cardiac function, equipment conditions, or operator experience can shift the scan away from the arterial phase, as can a delayed repeat acquisition in a patient who cannot cooperate (9, 10). The resulting images lack arterial enhancement adequate for vascular interpretation, a situation we refer to as arterial-phase acquisition failure. Approximately 1.8% of conventional CTA examinations have been reported as inadequate for evaluating the intracranial arteries because of unsatisfactory arterial-phase opacification (11). Such failures are likely more frequent in primary hospitals and interhospital transfer settings, where individualized injection protocols and experienced technologists may be unavailable (12). Once acquisition fails, patients often need a second iodinated contrast injection and a repeat scan, or experience delayed transfer, all of which cost time within the thrombectomy window.

Previous approaches to improving inadequate vascular depiction on CTA have not directly addressed arterial-phase acquisition failure. CTP-derived CTA reconstruction requires a complete dynamic acquisition (13, 14), whereas deep-learning-based CTA denoising, vessel enhancement, and low-dose or low-iodine restoration mainly improve arterial-phase images that have already been acquired, rather than recovering vascular enhancement that was not captured (15–17). These methods are also mostly optimized and evaluated using pixel-level fidelity metrics, which do not necessarily align with clinical interpretability (18). A complementary line of research, including CNN-TriFuseResNet-50 and M^2^FNet, has investigated direct detection of large-vessel occlusion from non-contrast CT (19, 20). Unlike direct detection methods that output an occlusion classification, RescueCTA generates vascular images that provide visual information about vessel course and occlusion location to support reader interpretation of underopacified CTA. Developing such a generative restoration model is constrained by the scarcity of paired data, as the same patient rarely undergoes both underopacified and adequately opacified CTA. Late-frame CT perfusion (CTP) therefore provided a standardized surrogate for constructing within-subject image pairs. However, late-frame CTP is not equivalent to genuinely underopacified CTA because of differences in acquisition protocols, noise characteristics, and image reconstruction. Accordingly, same-session late-frame CTP–CTA pairs were used for model training and primary evaluation, whereas genuinely underopacified CTA was evaluated separately in the exploratory cohort.

We therefore developed RescueCTA, a rescue CTA image-synthesis model based on a 2.5D conditional generative adversarial network (GAN). RescueCTA images are intended as supplementary imaging after acquisition failure, not as a replacement for a clinically repeated CTA. We hypothesized that reading them alongside the underopacified images would improve the detection of proximal anteriorcirculation target LVO. Model development and the primary evaluation used last-frame CTP images as source images to simulate acquisition failure. In addition, we evaluated the model in a small exploratory cohort of truly underopacified CTA.

## 2 Materials and methods

### 2.1 Study cohorts and image processing

For model development and primary evaluation, we included patients who underwent head CTA and CTP in a single examination for suspected acute ischemic stroke (AIS) at our institution between December 2022 and December 2023. In these patients, the CTA of the same session served as the paired CTA. We excluded intracerebral hemorrhage, prior craniotomy, traumatic intracerebral hematoma, examinations that were noninterpretable for technical reasons, and cases in which CTP could not be adequately registered to CTA. Inadequate arterial opacification itself was not an exclusion, as such images were the target of the study. These patients were split at the patient level, with training and validation cases forming the model-development cohort and the remainder forming the primary-evaluation cohort.

We also included patients with suspected AIS between June 2018 and March 2024 who had a genuinely underopacified CTA and, within 2 days, a paired CTA with adequate opacification, identified by manual picture archiving and communication system (PACS) search. Such cases typically arose when a first acquisition failed because the patient could not cooperate and the immediate repeat scan also missed the arterial phase; the resulting underopacified series served as the source image. Because the intracranial arteries could not be assessed reliably enough for routine clinical decision-making, a further CTA was required, which served as the paired CTA. These patients formed the exploratory evaluation cohort. The paired CTA was obtained a median of 26 h 44 min (interquartile range [IQR], 25 h 02 min to 28 h 38 min) after the underopacified CTA and used a separate contrast injection, so vascular status may have changed between the two examinations. The selection flowchart is shown in Figure 1.

**Figure 1:**
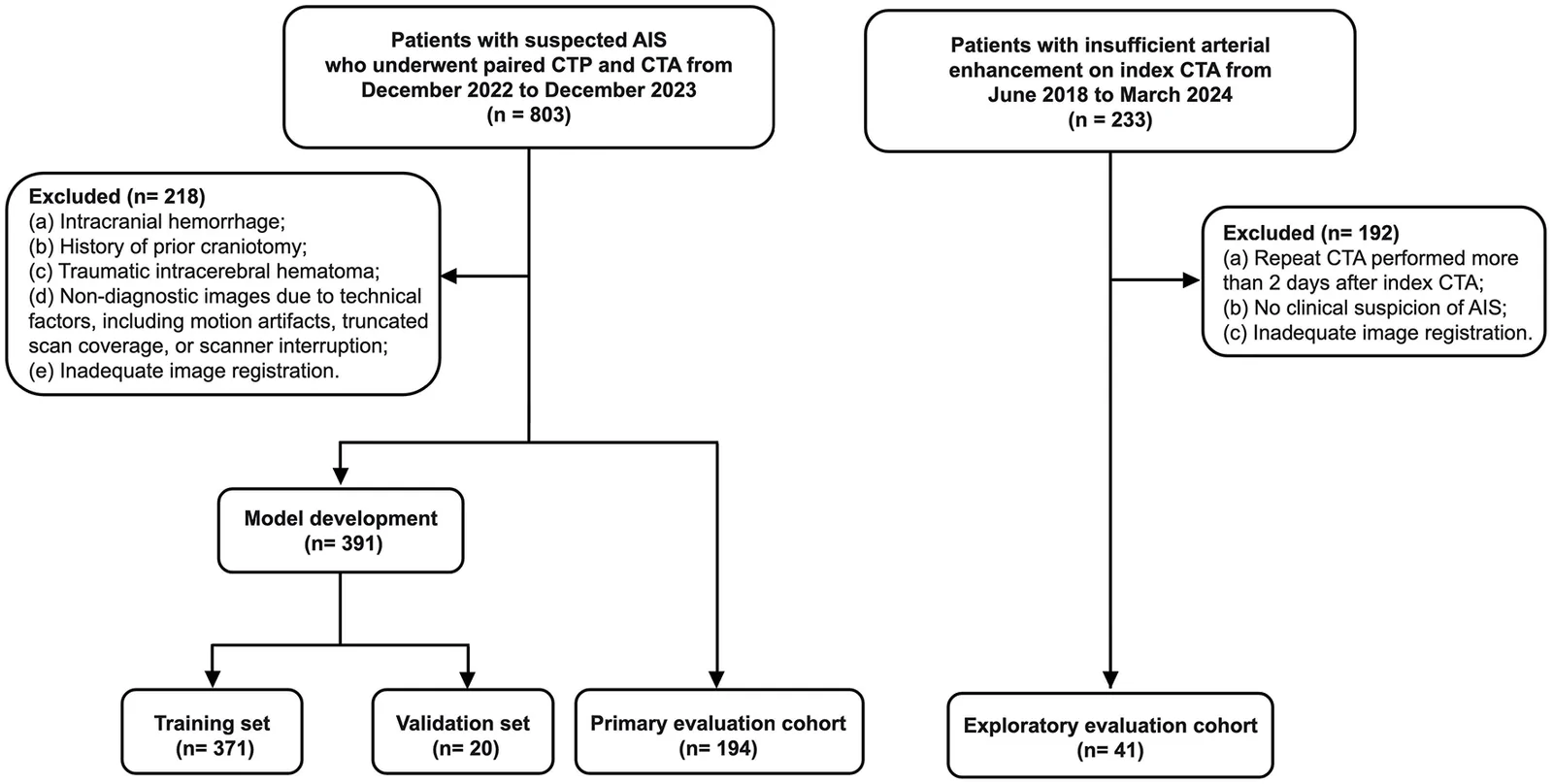
Patient Selection and Cohort Allocation. Among 803 patients with suspected acute ischemic stroke who underwent paired CT perfusion (CTP) and CT angiography (CTA), 218 were excluded, and 585 were retained; 391 were assigned to model development (371 for training and 20 for validation), and 194 formed the primary evaluation cohort. An independent search identified 233 patients with insufficient arterial enhancement on the index CTA; after 192 exclusions, 41 formed the exploratory evaluation cohort. Detailed exclusion criteria are shown in the flow diagram. AIS, acute ischemic stroke; CTA, CT angiography; CTP, CT perfusion.

CTA and CTP for the model-development and primary-evaluation cohorts were acquired on a United Imaging Healthcare uCT 960+, both at 100 kVp with 1-mm sections, helical for CTA (165–177 mAs) and axial for CTP (69–74 mAs per frame), in the same session after a single contrast injection. Dynamic CTP comprised 21–22 frames, with the last acquired approximately 57–62 s after the first; this last-frame CTP image was used as the simulated acquisition-failure input, and its arterial enhancement is compared with that of truly underopacified CTA in Supplementary Figure S1. CTA in the exploratory evaluation cohort was acquired on a Philips iCT 256 or Siemens SOMATOM Force, providing cross-vendor evaluation. Full acquisition parameters, contrast protocols, and dose data are given in Supplementary Table S1. For paired training-data preparation only, CTA was cropped to the CTP field of view, and CTP was registered to CTA space with the symmetric normalization (SyN) algorithm implemented in Advanced Normalization Tools (ANTs), using CTA as the fixed image (21, 22). Registrations with a foreground-mask Dice coefficient < 0.85 were excluded. Images in the training, validation, and test sets were resampled to 512 × 512 with 1-mm sections, clipped to -1024 to 3071 Hounsfield units (HU), normalized to [-1, 1], and grouped into 2.5D three-channel inputs of three adjacent sections.

### 2.2 Model development

RescueCTA was developed as a paired, supervised 2.5D conditional generative adversarial network to synthesize a rescue CTA volume from a single-phase underopacified acquisition (Figure 2). For each axial position, three consecutive source sections were supplied as a three-channel input, and the corresponding registered CTA sections served as targets. Source and target volumes were cropped using the same head bounding box and resized to 512 × 512. During inference, only the central channel from each three-section output was retained, and the resulting sections were stacked to reconstruct the three-dimensional volume.

**Figure 2:**
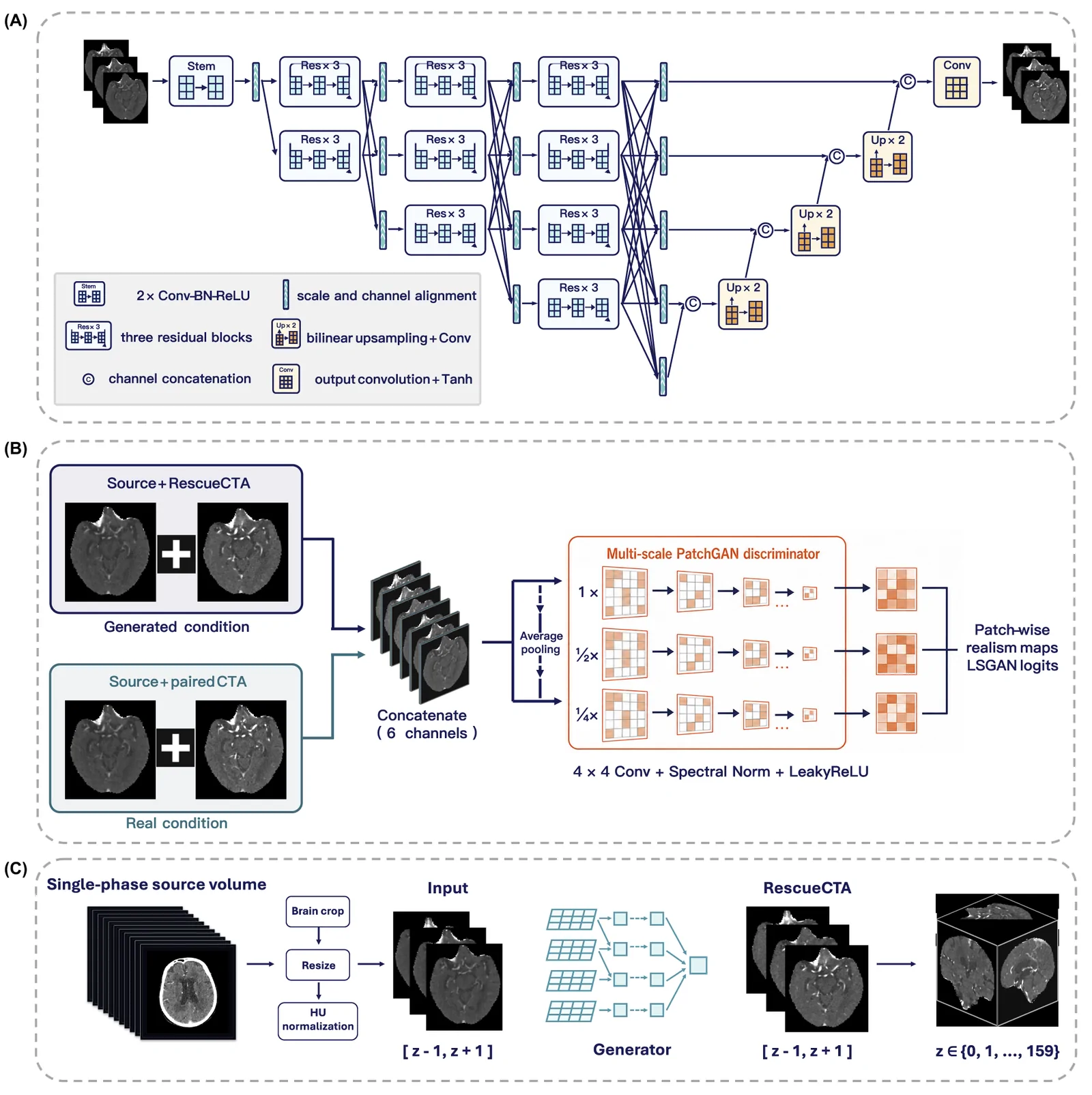
RescueCTA Model Architecture and Inference Pipeline. Architecture and inference pipeline of the 2.5D conditional generative adversarial network. **(A)** Generator: parallel branches at several resolutions, built from residual blocks and linked by repeated multi-resolution fusion, are merged by channel concatenation before the output convolution. **(B)** Discriminator: the source image is concatenated with either the RescueCTA image or the paired CTA to form a 6-channel input, which a conditional multiscale PatchGAN evaluates at full, one-half, and one-quarter resolutions, each yielding a patch-wise realism map. **(C)** Inference: from a single-phase source volume, after brain cropping, resizing, and HU normalization, three consecutive sections (z - 1 to z + 1) are passed to the generator, which outputs the RescueCTA image of the central section; sections are then stacked over z to reconstitute the volume. CTA, CT angiography; HU, Hounsfield unit.

The generator used a high-resolution network (HRNet) backbone (23, 24) with parallel multiresolution branches and repeated cross-scale fusion. This design preserved a full-resolution feature stream for small-vessel detail while incorporating contextual information from lower-resolution branches. A spectrally normalized conditional PatchGAN discriminator (25, 26) evaluated source–target pairs at three image scales. The generator was optimized with a weighted combination of L1 reconstruction, VGG-19 perceptual (27), feature-matching, edge, and Laplacian losses. The edge and Laplacian terms were introduced progressively during later training to refine vessel boundaries while maintaining optimization stability.

Training was conducted on a Linux platform using eight NVIDIA A100 graphics processing units (GPUs), each with 80 GB of memory. The complete architecture, loss-weight schedule, optimization settings, and inference code are available at https://github.com/Becomingw/RescueCTA.

### 2.3 Quantitative evaluation

RescueCTA was compared with Pix2Pix (28), Pix2PixHD (25), and CycleGAN (29), each trained from its official public implementation using the same preprocessing, 2.5D input, data split, and training schedule as RescueCTA. Similarity to the paired CTA was measured by normalized mean squared error (nMSE), peak signal-to-noise ratio (PSNR), and structural similarity index measure (SSIM) (30), computed in vascular (window width/level 400/50), soft-tissue (400/40), and bone (2000/400) windows. Additionally, SSIM, PSNR, and nMSE were calculated within vessel segmentation masks.

### 2.4 Visual quality evaluation

Two radiologists with 10 years of experience independently rated the paired CTA and each model output in both evaluation cohorts. Versions were anonymized and presented side by side in random order, and readers had no access to model identity, quantitative results, or each other's scores. Overall image quality and artifacts were each rated on a 5-point scale (31). For overall image quality: (1) completely unusable for diagnosis or failed generation; (2) poor image quality that may impair diagnosis; (3) basically usable but with limited diagnostic confidence; (4) good image quality, usable for diagnosis; and (5) excellent image quality with continuous, clearly depicted vessels, usable for diagnosis. For image artifacts: (1) severe artifacts that markedly impair diagnosis; (2) fairly conspicuous artifacts that may affect diagnostic judgment; (3) mild-to-moderate artifacts, but the image remains basically usable for diagnosis; (4) only minimal artifacts that do not affect diagnosis; and (5) no appreciable artifacts, clear image. A score of 3 or higher was defined as acceptable image quality. Disagreements were resolved by discussion into a case-level consensus score.

### 2.5 Diagnostic evaluation

Readers judged the presence of target LVO, defined as terminal internal carotid artery occlusion or M1 segment occlusion of the middle cerebral artery; M2 occlusion, M1 stenosis without occlusion, atherosclerotic change, and normal vessels were classified as negative. The composite reference standard was established independently by two neuroradiologists with more than 15 years of experience who did not take part in the reading, with a third neuroradiologist, also with more than 15 years of experience, adjudicating any disagreements. They could review the clinically acquired CTA, noncontrast CT, CTP, digital subtraction angiography (DSA), operative or thrombectomy records, and clinical follow-up, but not RescueCTA images, other model outputs, or reader results. All reference-standard-positive cases were confirmed by DSA or thrombectomy records.

In the primary evaluation cohort, four readers who regularly interpret stroke CTA took part, none of whom had rated image quality: readers 1 and 2 had 3 to 5 years of experience, and readers 3 and 4 had 8 to 10 years of experience. Under a multireader multicase (MRMC) design, they compared reading last-frame CTP images alone with combined reading of last-frame CTP images plus RescueCTA images (32). Reading was split into sessions at least 4 weeks apart, with each case shown under only one condition per session, and the paired CTA was read 4 weeks after the main study. Images were anonymized and randomized, and readers were blinded to the reference standard, to other readers, and to clinical information. Each reading recorded image interpretability, the suspected side of occlusion, and a 1–5 diagnostic confidence score (1, definitely no target LVO; 3, indeterminate; and 5, definite target LVO). A reading was recorded as noninterpretable when image quality precluded vascular assessment, that is, when the intracranial arteries showed no discernible opacification or when noise prevented the vessels from being followed. Readers 3 and 4 repeated the same procedure in the exploratory evaluation cohort.

### 2.6 Statistical analysis

Image quality analyses used case-level consensus scores, and acceptable rates are reported descriptively. Distributions across the five image conditions were compared by the Friedman test, with prespecified two-sided Wilcoxon signed-rank comparisons of RescueCTA images against the paired CTA, Pix2Pix, Pix2PixHD, and CycleGAN images. Zero differences were handled by the Pratt method, and Holm correction was applied within each score domain.

The primary analysis compared areas under the receiver operating characteristic (ROC) curves (AUCs) for reading last-frame CTP images alone and for combined reading, derived from the 1–5 confidence scores. The paired CTA was analyzed identically as a reference condition but was not treated as the reference standard. Noninterpretable readings were excluded from this analysis. AUCs were compared by MRMC analysis in the R package MRMCaov 0.3.1 (33, 34), using empirical AUC and an unbiased covariance estimator for the standard error, 95% confidence interval (CI), and two-sided *p*-value of the between-condition difference.

Binary performance used a clinical-failure definition, in which scores of 4–5 were positive, scores of 1–2 were negative, and noninterpretable readings and scores of 3 (indeterminate) were counted as errors according to the direction of the reference standard. A true positive required both a positive judgment and the correct identification of the side of occlusion; positive judgments with the wrong side were counted as false negatives. Sensitivity, specificity, accuracy, balanced accuracy, and the noninterpretable rate are reported for all conditions, with Cohen κ for interreader agreement. Binary metrics were compared between reading conditions by the exact McNemar test and balanced accuracy by a paired bootstrap test; these comparisons were secondary and are reported without correction for multiplicity. Correct-side AUC was computed by assigning the lowest confidence score only to reference-standard-positive readings that were judged positive but with the wrong side of occlusion. Strict AUC combined the clinical-failure penalty for noninterpretable and indeterminate readings with the correct-side penalty above, without repenalizing false-negative readings for laterality. Confidence intervals and *p*-values for both were obtained from a paired stratified case bootstrap. Results in the exploratory evaluation cohort are descriptive only. Tests were two-sided, with p < 0.05 considered significant.

## 3 Results

### 3.1 Patient characteristics

A total of 803 patients with paired CTP and CTA were assessed for eligibility; 218 were excluded according to prespecified clinical, image-quality, or registration criteria. The remaining 585 patients (median age, 73 years [IQR, 66–81]; 380 men [65.0%], 205 women [35.0%]) were analyzed. Of these, 371 patients (42,595 paired 2.5D sections) were used for training and 20 (2,315 pairs) for validation, together forming the model-development cohort, and the remaining 194 formed the primary-evaluation cohort. The exploratory evaluation cohort was assembled by an independent manual PACS search: of 233 underopacified CTA examinations, 192 were excluded because the paired CTA exceeded 2 days or the clinical records did not support suspected AIS, leaving 41 patients. Cohort characteristics are given in Table 1.

**Table 1:** Characteristics of the evaluation cohorts.

Characteristic	Primary evaluation cohort	Exploratory evaluation cohort
No. of patients	194	41
Age, y, median [IQR]	71.5 [65.0, 78.0]	68.0 [60.0, 71.0]
Male, n/N (%)	129/194 (66.5%)	17/41 (41.5%)
Hypertension, n/N (%)	123/194 (63.4%)	25/41 (61.0%)
Diabetes mellitus, n/N (%)	32/194 (16.5%)	7/41 (17.1%)
Hyperlipidemia, n/N (%)	68/194 (35.1%)	12/41 (29.3%)
Atrial fibrillation, n/N (%)	41/194 (21.1%)	2/41 (4.9%)
Admission NIHSS score, median [IQR]	4.0 [2.0, 11.0]	0.0 [0.0, 1.0]
Interval from the source CTA to the paired CTA, hh:mm:ss, median [IQR]	NA	26:44:25 [25:02:28, 28:38:10]
Intravenous thrombolysis, n/N (%)	34/194 (17.5%)	0/41 (0.0%)
Endovascular thrombectomy, n/N (%)	61/194 (31.4%)	2/41 (4.9%)
Discharge NIHSS score, median [IQR]	2.0 [0.8, 6.0]	0.0 [0.0, 1.0]
Creatinine, μmol/L, median [IQR]	73.5 [63.4, 96.0]	64.7 [55.6, 79.8]
eGFR, mL/min/1.73 m^2^, median [IQR]	81.1 [61.6, 90.3]	88.6 [77.6, 96.1]
Reference-standard target LVO, n/N (%)	61/194 (31.4%)	2/41 (4.9%)
Terminal internal carotid artery occlusion, n/N (%)	10/194 (5.2%)	0/41 (0.0%)
M1-segment occlusion, n/N (%)	51/194 (26.3%)	2/41 (4.9%)
M2-segment occlusion, n/N (%)	25/194 (12.9%)	2/41 (4.9%)
Severe M1 stenosis, n/N (%)	39/194 (20.1%)	7/41 (17.1%)
Moderate M1 stenosis, n/N (%)	18/194 (9.3%)	2/41 (4.9%)
Mild M1 stenosis, n/N (%)	3/194 (1.5%)	0/41 (0.0%)
Atherosclerosis, n/N (%)	44/194 (22.7%)	27/41 (65.9%)
Normal, n/N (%)	4/194 (2.1%)	1/41 (2.4%)

Unless otherwise indicated, continuous variables are presented as median [IQR] and categorical variables as n/N (%). Target LVO was defined as terminal internal carotid artery occlusion or M1-segment occlusion. In two patients, the repeat CTA was obtained immediately, within the same examination and after a second contrast injection; the reported median and IQR include these two patients. CTA, CT angiography; IQR, interquartile range; NIHSS, National Institutes of Health Stroke Scale; eGFR, estimated glomerular filtration rate; LVO, large-vessel occlusion; NA, not applicable.

### 3.2 Quantitative evaluation

Quantitative results are shown in Table 2. Model rankings were not consistent across cohorts, display windows, and metrics. In the vascular window of the primary-evaluation cohort, RescueCTA achieved an SSIM of 0.915 (95% CI, 0.914–0.917), a PSNR of 30.773 dB (95% CI, 30.496–31.026), and an nMSE of 0.027 (95% CI, 0.024–0.030). Pix2Pix achieved the highest SSIM (0.924 [95% CI, 0.922–0.926]), whereas Pix2PixHD achieved the highest PSNR (32.264 dB [95% CI, 31.781–32.698]) and the lowest nMSE (0.024 [95% CI, 0.020–0.029]). In the bone window, RescueCTA and Pix2Pix achieved the highest SSIM (both 0.989), RescueCTA achieved the lowest nMSE (0.008 [95% CI, 0.006–0.010]), and Pix2PixHD achieved the highest PSNR (44.153 dB [95% CI, 43.247–45.013]). Soft-tissue-window values for both evaluation cohorts are given in Supplementary Table S2. In the vessel-mask analysis, RescueCTA achieved the highest SSIM (0.8021) and PSNR (25.3729 dB) and the lowest nMSE (2.4383), whereas Pix2Pix showed the lowest SSIM (0.6321) and PSNR (12.7568 dB) and the highest nMSE (39.3831) (Supplementary Table S3). In the exploratory evaluation cohort, RescueCTA achieved a vascular-window SSIM of 0.921 (95% CI, 0.911–0.930), a PSNR of 28.840 dB (95% CI, 28.177–29.461), and an nMSE of 0.041 (95% CI, 0.038–0.045). A component ablation analysis of the generator, discriminator, perceptual/feature-matching losses, and progressive loss schedule is provided in Supplementary Table S8.

**Table 2:** Quantitative image quality by cohort and display window.

Window	Model	SSIM ↑	PSNR (dB) ↑	nMSE ↓
Primary evaluation cohort				
Vascular	**RescueCTA**	0.915 [0.914–0.917]	30.773 [30.496–31.026]	0.027 [0.024–0.030]
	Pix2Pix	**0.924 [0.922–0.926]**	31.631 [31.202–32.023]	0.025 [0.022–0.029]
	Pix2PixHD	0.911 [0.908–0.913]	**32.264 [31.781–32.698]**	**0.024 [0.020–0.029]**
	CycleGAN	0.907 [0.904–0.910]	30.619 [30.043–31.138]	0.047 [0.034–0.064]
Bone	**RescueCTA**	**0.989 [0.988–0.990]**	42.803 [42.112–43.449]	**0.008 [0.006–0.010]**
	Pix2Pix	**0.989 [0.988–0.990]**	43.179 [42.330–43.965]	0.012 [0.008–0.016]
	Pix2PixHD	0.987 [0.985–0.988]	**44.153 [43.247–45.013]**	0.012 [0.008–0.016]
	CycleGAN	0.984 [0.982–0.987]	42.593 [41.768–43.357]	0.016 [0.009–0.024]
Exploratory evaluation cohort				
Vascular	**RescueCTA**	0.921 [0.911–0.930]	28.840 [28.177–29.461]	0.041 [0.038–0.045]
	Pix2Pix	**0.933 [0.923–0.941]**	29.594 [28.939–30.243]	0.035 [0.032–0.038]
	Pix2PixHD	0.920 [0.908–0.931]	30.132 [28.966–31.180]	0.039 [0.027–0.056]
	CycleGAN	0.921 [0.912–0.930]	**31.550 [30.755–32.294]**	**0.023 [0.020–0.026]**
Bone	**RescueCTA**	0.990 [0.988–0.991]	42.236 [41.599–42.857]	0.005 [0.004–0.005]
	Pix2Pix	**0.991 [0.989–0.992]**	42.627 [42.058–43.171]	**0.004 [0.004–0.005]**
	Pix2PixHD	0.986 [0.980–0.990]	42.548 [41.133–43.755]	0.009 [0.004–0.016]
	CycleGAN	0.988 [0.986–0.990]	**43.474 [42.465–44.342]**	0.005 [0.003–0.007]

Values are means [bootstrap 95% CIs]. ↑, higher is better; ↓, lower is better. Bold and underlined values denote the best and second-best means, respectively. Vascular and bone windows were WW/WL 400/50 and 2000/400. CI, confidence interval; nMSE, normalized mean squared error; PSNR, peak signal-to-noise ratio; SSIM, structural similarity index measure; WW/WL, window width/window level.

### 3.3 Visual quality evaluation

Subjective scores are shown in Figure 3. In the primary-evaluation cohort, the overall image quality of RescueCTA images was 3.90 ± 0.81, acceptable in 92.3% (179/194), above Pix2PixHD (3.47 ± 0.92; 90.7% [176/194]), Pix2Pix (2.39 ± 0.80; 44.3% [86/194]), and CycleGAN (1.87 ± 1.03; 19.6% [38/194]), and below the paired CTA (4.72 ± 0.57; 99.5% [193/194]). Artifact scores were 4.79 ± 0.66 for RescueCTA images (97.9% [190/194]), 4.71 ± 0.87 for Pix2Pix (93.8% [182/194]), 4.70 ± 0.91 for Pix2PixHD (94.8% [184/194]), 4.66 ± 1.02 for CycleGAN (92.3% [179/194]), and 4.96 ± 0.28 for the paired CTA (99.5% [193/194]). Both domains differed across the five conditions (Friedman χ^2^ = 575.01 and 45.66, degrees of freedom [df] = 4; both p < 0.001). In Holm-corrected Wilcoxon tests, RescueCTA images scored below the paired CTA and above all three baselines in both domains (all corrected p < 0.001, except artifacts vs. Pix2PixHD, corrected *p* = 0.004; Supplementary Table S4). In the exploratory-evaluation cohort, RescueCTA images scored 3.51 ± 1.12 for overall quality (78.0% [32/41]) and the paired CTA 4.17 ± 0.83 (95.1% [39/41]); both were rated free of artifacts in every case (5.00 ± 0.00).

**Figure 3:**
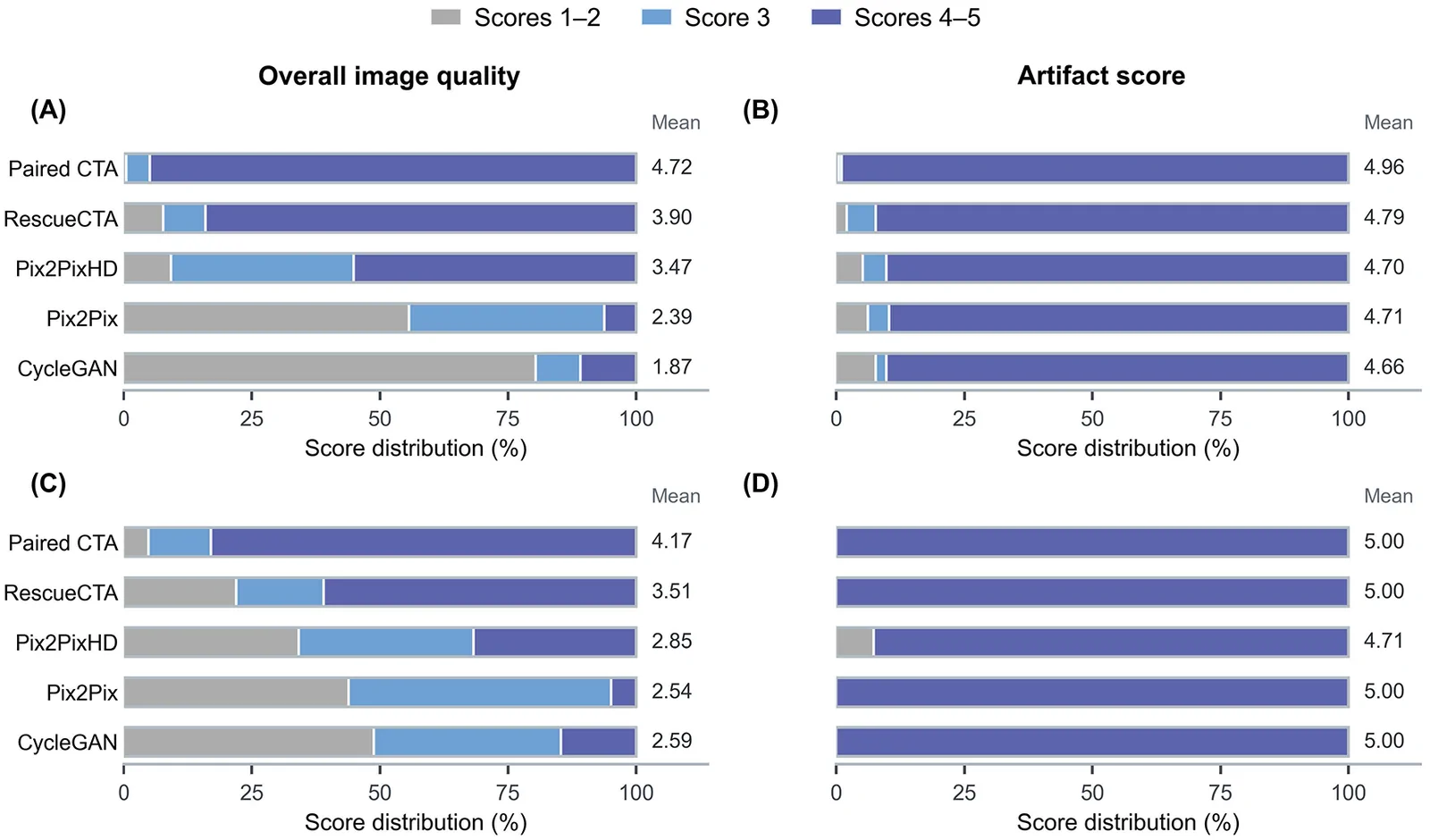
Visual Quality of RescueCTA and the Baseline Models. **(A, B)** Distributions of overall image quality scores **(A)** and artifact scores **(B)** in the primary evaluation cohort. **(C, D)** Corresponding score distributions in the exploratory evaluation cohort. Image quality scores are case-level consensus scores reached after discussion between the two readers. Bars show the proportions of cases with scores of 1–2, 3, and 4–5; numbers at the right show mean scores.

Figure 4 and Supplementary Figures S2, S3 show representative image appearances and artifact patterns across the models. Their appearance paralleled the subjective scores: on axial images, RescueCTA and Pix2PixHD images both showed good vascular clarity, but the granular noise and interslice discontinuity of Pix2PixHD were conspicuous on coronal and sagittal reformats; Pix2Pix and CycleGAN showed even more pronounced artifacts. Supplementary Figure S4 shows two RescueCTA failure cases in which some vessels visible on paired CTA were not fully reconstructed.

**Figure 4:**
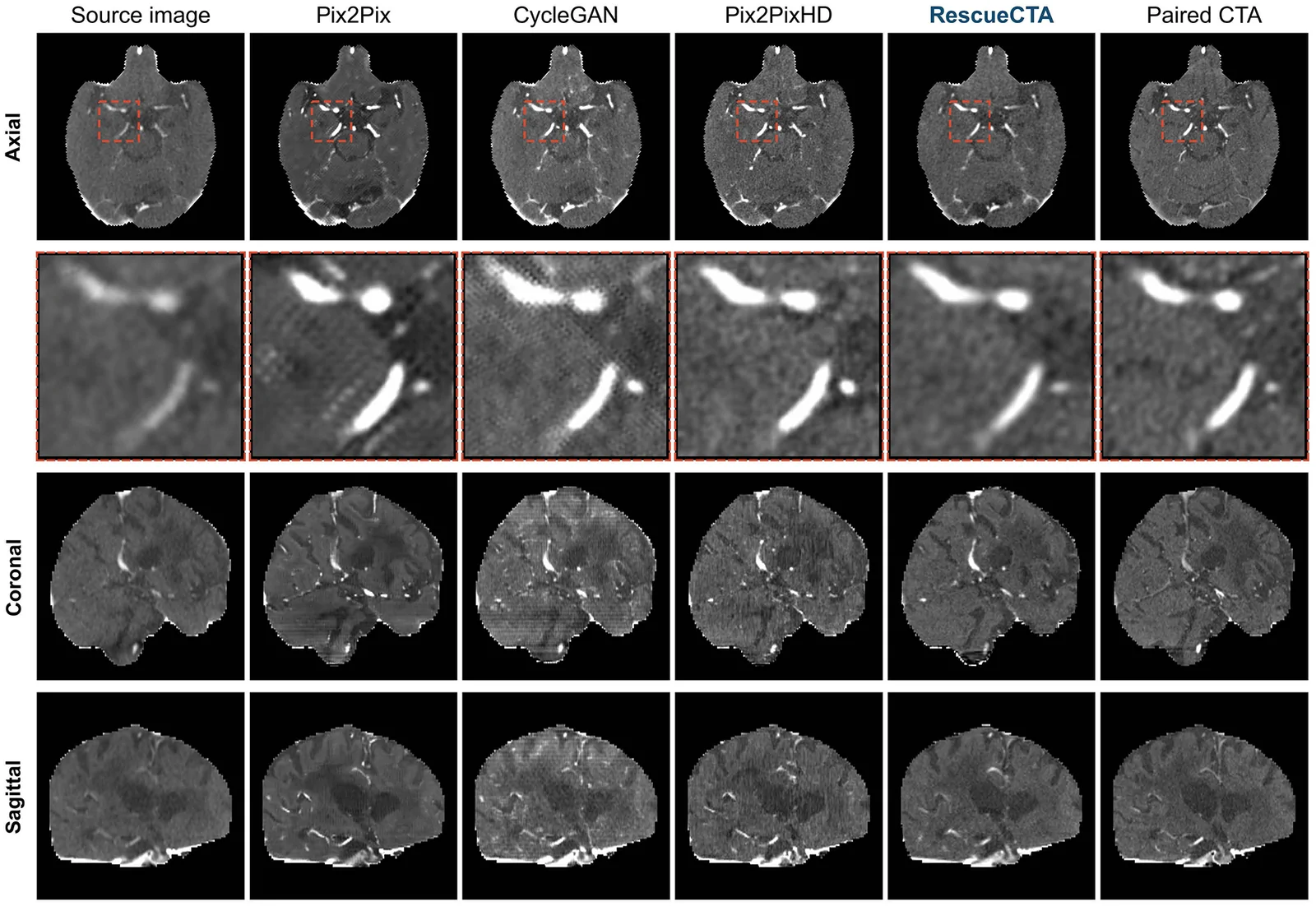
Representative Qualitative Comparison. A representative case. The first row shows full axial images, with the matched region of interest outlined by a red dashed box; the second row shows magnified views of the same region; the third and fourth rows show coronal and sagittal images of the same case, respectively. All images are displayed at the same window width/window level.

### 3.4 Diagnostic evaluation

Diagnostic results are shown in Table 3 and Figure 5. Combined reading of last-frame CTP images plus RescueCTA images gave a higher AUC than last-frame CTP images alone (0.933 [95% CI, 0.887–0.978] vs. 0.850 [95% CI, 0.765–0.934]; ΔAUC = 0.083 [95% CI, 0.025–0.141]; *p* = 0.007). The paired CTA reference condition reached 0.948 [95% CI, 0.913–0.982], numerically higher than combined reading but not significantly so (ΔAUC = 0.015 [95% CI, –0.043 to 0.073]; *p* = 0.594). In the strict-AUC sensitivity analysis, which penalized non-interpretable and indeterminate readings and wrong-side positive judgments, combined reading remained superior (0.880 [95% CI, 0.838–0.918] vs. 0.756 [95% CI, 0.692–0.815]; ΔAUC = 0.124 [95% CI, 0.069–0.181]; *p* < 0.001), and the paired CTA reached 0.935 [95% CI, 0.901–0.965], higher than combined reading (ΔAUC = 0.055 [95% CI, 0.011–0.100]; *p* = 0.019). In the component analyses, the failure-penalized AUCs were 0.782, 0.891, and 0.944, and the correct-side AUCs were 0.820, 0.920, and 0.939 for last-frame CTP images alone, combined reading, and the paired CTA, respectively (Supplementary Figure S5).

**Table 3:** Diagnostic performance for target LVO diagnosis in the primary evaluation cohort.

Panel A. Discrimination			
Metric	Last-frame CTP alone	Last-frame CTP + RescueCTA	Paired CTA
AUC (95% CI)	0.850 (0.765–0.934)	0.933 (0.887–0.978)	0.948 (0.913–0.982)
Strict AUC (95% CI)	0.756 (0.692–0.815)	0.880 (0.838–0.918)	0.935 (0.901–0.965)
Panel B. Clinical performance			
Sensitivity	140/244 (57.4%)	197/244 (80.7%)	218/244 (89.3%)
Specificity	459/532 (86.3%)	475/532 (89.3%)	500/532 (94.0%)
Accuracy	599/776 (77.2%)	672/776 (86.6%)	718/776 (92.5%)
Balanced accuracy	71.8%	85.0%	91.7%
Non-interpretable rate	5.0% (39/776)	3.1% (24/776)	0.3% (2/776)

Except for balanced accuracy, binary metrics are shown as n/N (%). Reader-level results are provided in Figure 6 and Supplementary Table S7. AUC, area under the receiver operating characteristic curve; CI, confidence interval; CTP, CT perfusion; LVO, large-vessel occlusion.

**Figure 5:**
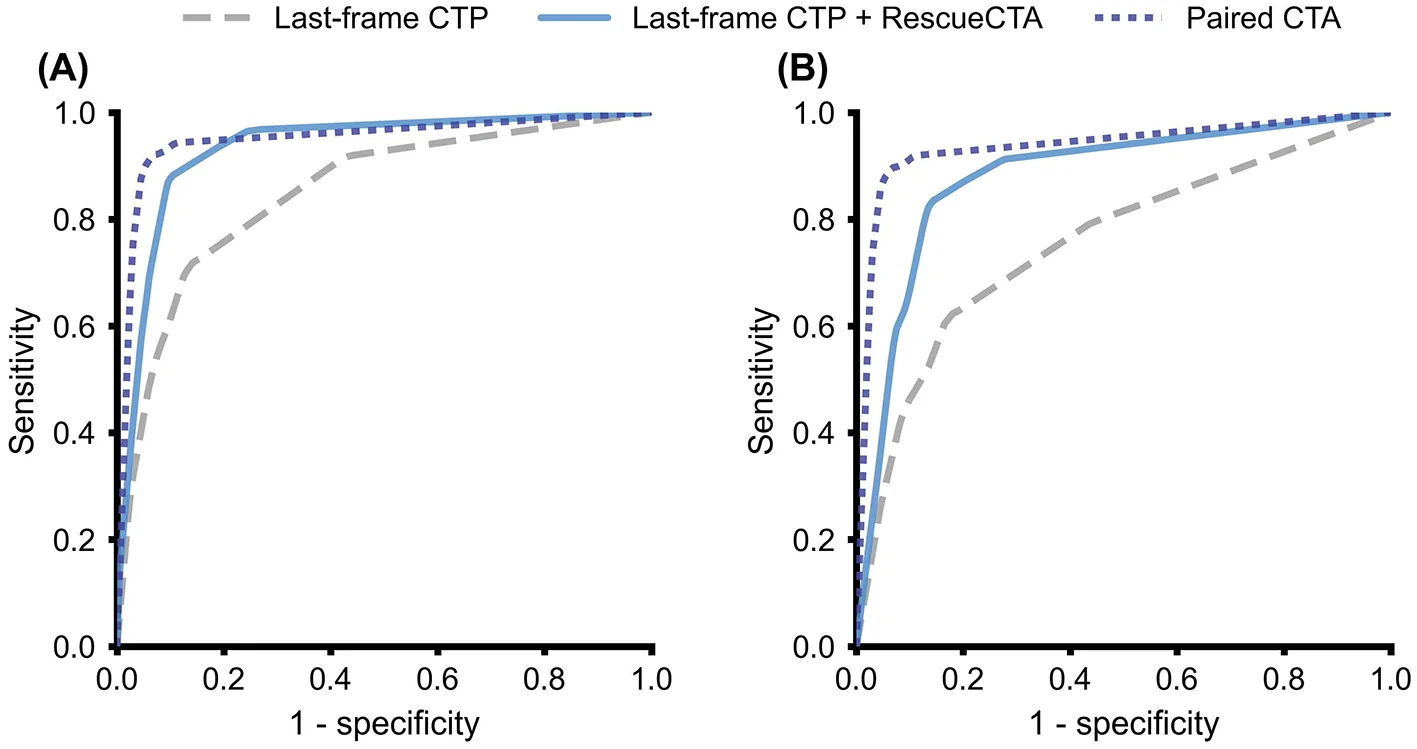
Diagnostic Performance in the Primary Evaluation Cohort. **(A)** Reader-averaged ROC curves for target large-vessel occlusion diagnosis in the primary evaluation cohort. Gray, blue, and indigo curves denote reading last-frame CTP images alone, last-frame CTP images + RescueCTA, and the paired CTA, respectively. AUCs were computed from the raw 1–5 diagnostic confidence scores. **(B)** Strict-AUC sensitivity analysis, in which non-interpretable and indeterminate readings were counted as errors and, in reference-standard-positive cases, positive judgments identifying the wrong side were also counted as errors. AUC, area under the receiver operating characteristic curve; CTA, CT angiography; CTP, CT perfusion; ROC, receiver operating characteristic.

The improvement was directionally consistent across readers, with all 4 achieving a higher AUC, strict AUC, sensitivity, and accuracy under combined reading; under the strict definition, the gain was largest in the two readers with 3 to 5 years of experience (Figure 6; Supplementary Figure S6). Sensitivity, specificity, and accuracy were 80.7, 89.3, and 86.6% for combined reading; 57.4, 86.3, and 77.2% for last-frame CTP images alone; and 89.3, 94.0, and 92.5% for the paired CTA. Balanced accuracy rose from 71.8 to 85.0, vs. 91.7% for the paired CTA. Between the two readers with 3 to 5 years of experience, interreader agreement improved from κ = 0.687 to 0.897, reaching that of the two readers with 8 to 10 years of experience under the same condition (0.882), whereas the latter changed from 0.855 to 0.882 (Supplementary Table S5).

**Figure 6:**
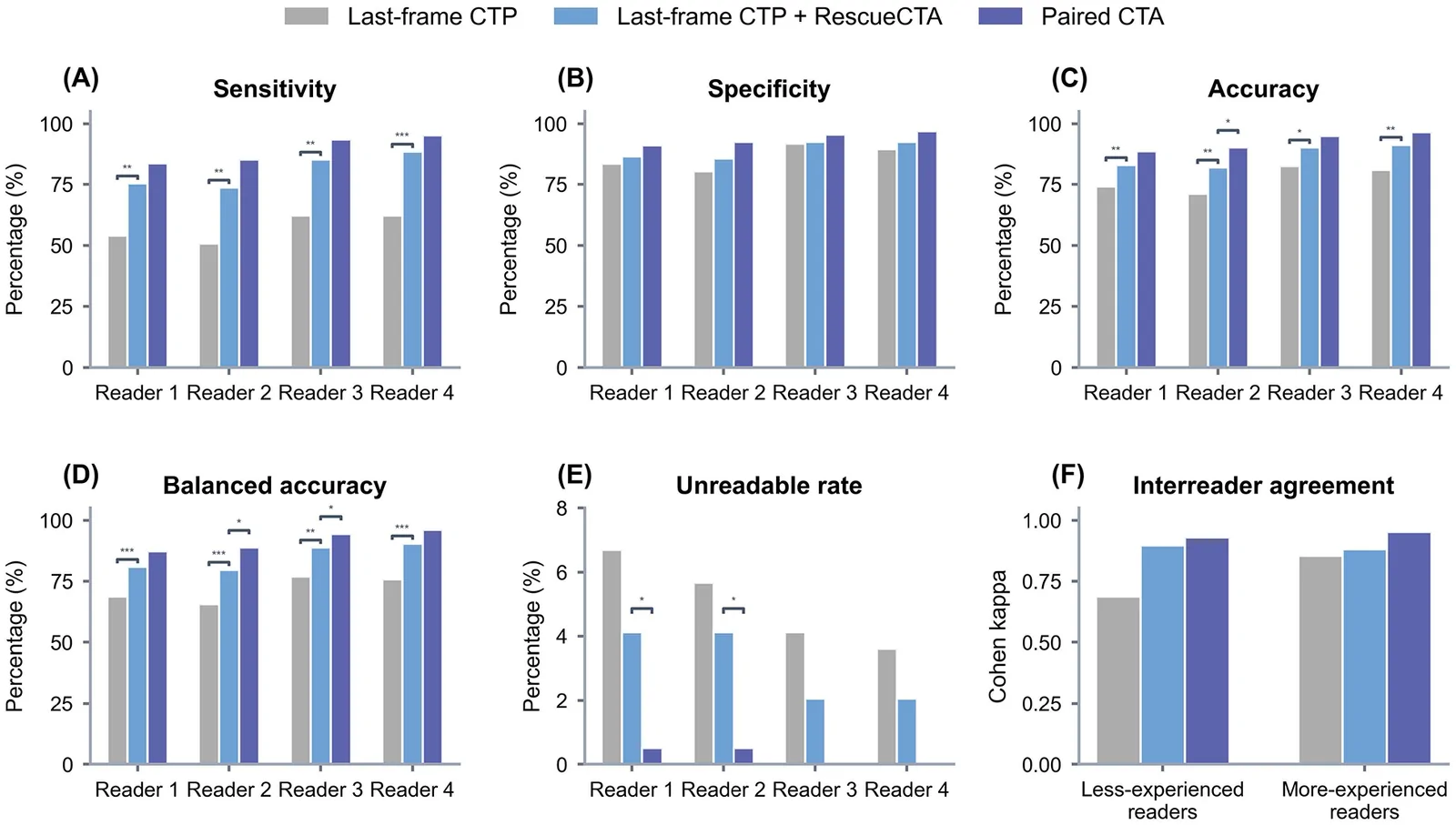
Reader-Level Diagnostic Performance in the Primary Evaluation Cohort. **(A–E)** Sensitivity **(A)**, specificity **(B)**, accuracy **(C)**, balanced accuracy **(D)**, and non-interpretable rate **(E)** for target large-vessel occlusion diagnosis by each reader in the primary evaluation cohort. Readers 1 and 2 have 3 to 5 years of experience, and readers 3 and 4 have 8 to 10 years of experience. **(F)** Binary diagnostic agreement stratified by reader experience. Binary metrics use the clinical-failure definition given in the Methods. Gray bars denote reading last-frame CTP images alone, blue bars denote combined reading of last-frame CTP images plus RescueCTA images, and indigo bars denote the paired CTA. Brackets indicate the paired comparisons displayed; asterisks denote two-sided *p*-values (*p* < 0.05, *p* < 0.01, *p* < 0.001). *P*-values for binary metrics were computed using the exact McNemar test, and those for balanced accuracy using a paired bootstrap test. CTA, CT angiography; CTP, CT perfusion.

Noninterpretable readings numbered 24/776 (3.1%) for combined reading, 39/776 (5.0%) for last-frame CTP images alone, and 2/776 (0.3%) for the paired CTA, and among positive judgments, the side of occlusion was incorrect in five of 202 readings for combined reading, 10 of 150 for last-frame CTP images alone, and four of 222 for the paired CTA. False positives fell under combined reading for severe M1 stenosis (17.9% [28/156] to 14.7% [23/156]), moderate M1 stenosis (15.3% [11/72] to 4.2% [3/72]), and atherosclerosis (10.2% [18/176] to 6.8% [12/176]), whereas misclassification of M2 occlusion rose from 16.0% (16/100) to 19.0% (19/100), vs. 15.0% (15/100) for the paired CTA, with corresponding paired CTA values of 9.0% (14/156), 0.0% (0/72), and 1.7% (3/176) (Supplementary Figure S7).

The exploratory-evaluation cohort comprised 41 patients, two of whom were reference-standard-positive. Compared with underopacified CTA alone, combined reading gave a higher AUC (0.981 vs. 0.889), accuracy (93.9% [77/82] vs. 86.6% [71/82]), sensitivity (100.0% [4/4] vs. 75.0% [3/4]), specificity (93.6% [73/78] vs. 87.2% [68/78]), and balanced accuracy (96.8 vs. 81.1%). The paired CTA reached 1.000 and 100.0% on all measures (Supplementary Table S6).

## 4 Discussion

RescueCTA was evaluated in two complementary settings of reduced arterial opacification. In the primary-evaluation cohort, last-frame CTP images served as standardized simulations of underopacified single-phase images, with the same patients' paired CTA images serving as references. In the exploratory evaluation cohort, the source images were genuinely underopacified CTA examinations paired with subsequently acquired, adequately opacified CTA examinations. The primary cohort enabled the evaluation of RescueCTA under a relatively standardized simulation of underopacification, whereas the exploratory cohort provided a preliminary assessment of feasibility in actual arterial-phase acquisition failure. In the primary cohort, the subjective image quality of RescueCTA images was superior to that of the three baseline models, and combined reading of last-frame CTP and RescueCTA images significantly improved target LVO detection compared with reading last-frame CTP images alone. Combined reading did not differ significantly from paired CTA in the primary AUC analysis, although paired CTA remained superior under the strict definition (*p* = 0.019). Thus, the AUC improvement in the primary cohort supports the diagnostic value of RescueCTA under simulated underopacification. The directionally consistent findings in the exploratory cohort provide preliminary evidence of feasibility in actual CTA acquisition failure, but the real-world diagnostic performance of RescueCTA requires further validation in larger cohorts of genuinely underopacified CTA.

Two complementary mechanisms likely underlay the diagnostic improvement. The primary AUC analysis excluded noninterpretable readings, yet combined reading still increased the AUC by 0.083 over last-frame CTP images alone (*p* = 0.007). RescueCTA therefore improved discrimination between cases with and without target LVO even among images that readers could already interpret. Under the strict AUC, which additionally penalized noninterpretable and indeterminate readings and positive judgments with incorrect laterality, the difference increased to 0.124 (p < 0.001), indicating an additional reduction in diagnostic failures. Reader-level results showed larger gains among the readers with fewer years of experience, with greater improvements in both strict AUC and interreader agreement than among the more experienced readers. RescueCTA thus improved not only lesion discrimination but also the determination of the occluded side and the overall interpretability of the examination.

Underopacified CTA may prompt repeat imaging, additional contrast material and radiation exposure, and delayed vascular assessment. Because the benefit of endovascular treatment is time-dependent (35, 36), waiting for repeat imaging may delay triage or transfer decisions. The potential clinical value of RescueCTA lies in providing supplementary vascular information from the single low-opacification acquisition already available after the initial examination. Unlike previous CTP-derived CTA methods, which reconstruct CTA-like images from a complete dynamic series (13, 14), RescueCTA requires only a single-phase low-opacification image. Its application to genuinely underopacified CTA in the exploratory cohort further indicates that deployment does not depend on the availability of dynamic CTP data. RescueCTA should nevertheless be regarded as an adjunct to the source images rather than a replacement for diagnostic-quality CTA or clinically necessary repeat imaging.

Pixel-level quantitative metrics and clinical visual assessment did not produce consistent model rankings. SSIM, PSNR, and nMSE average similarity or error across the entire image, most of which consists of brain parenchyma, whereas the diagnostic task depends on the relatively small number of voxels forming the arteries. Some baseline models therefore ranked favorably on individual quantitative metrics despite poorer subjective image quality, vascular continuity, or multiplanar appearance than RescueCTA. Pixel-level similarity alone may consequently be insufficient to characterize the clinical value of generative CTA enhancement and should be complemented by radiologist assessment and task-based diagnostic evaluation.

Error analysis further delineated the limitations of RescueCTA in clinical interpretation. Although combined reading reduced false-positive classifications related to severe or moderate M1 stenosis and atherosclerotic changes, the proportion of M2 occlusions misclassified as target LVO increased from 16.0% with source-image reading alone to 19.0% with combined reading, compared with 15.0% using paired CTA. This finding indicates that concurrent review of the source images does not fully eliminate the risk of misleading information from RescueCTA. Incomplete reconstruction of distal branches may make the apparent vascular cutoff appear more proximal, leading readers to misclassify an M2 occlusion as a target LVO. Supplementary Figure S4 shows two representative failure cases in which some vessels visible on paired CTA were not fully reconstructed on RescueCTA, further illustrating that generated images may show vessel loss or discontinuity. No conspicuous artifacts were identified in the RescueCTA images in the exploratory cohort, although their overall image quality remained lower than that of paired CTA. Thus, outside the training conditions, incomplete vessel depiction may occur without an obvious image artifact. A visually plausible generated image does not ensure complete vascular information. This failure mode warrants particular caution when assessing distal branches and localizing occlusions, especially when inadequate opacification of the source images precludes confident determination of the occlusion level.

This study has several limitations. First, it was retrospective, and the model-development and primary-evaluation cohorts were drawn from the same scanner model. Generalizability across centers, scanners, and reconstruction protocols therefore remains to be established. Second, late-frame CTP was used as a standardized surrogate for underopacified CTA in the primary cohort. However, it is not equivalent to genuine CTA acquisition failure because the acquisition protocol, contrast distribution, noise characteristics, image reconstruction, and temporal information differ. The primary results should therefore be interpreted as evidence under simulated underopacification. Third, the exploratory evaluation cohort was small and included only two reference-standard-positive target LVO cases; its population composition also differed from that of an emergency target LVO cohort. This cohort therefore supports technical feasibility in genuinely underopacified CTA rather than a definitive estimate of diagnostic performance. Fourth, we evaluated image quality and diagnostic performance but did not directly assess the influence of RescueCTA on treatment selection, patient transfer, the need for repeat imaging, or clinical outcomes.

## Data Availability

The original contributions presented in the study are included in the article/Supplementary material, further inquiries can be directed to the corresponding author.
