# Supplementary material for: Generative enhancement of suboptimally opacified cerebral CT angiography for large-vessel occlusion detection: a multireader study
Source: Front Neurol. 2026 Oct 13;17:1955596. doi: 10.3389/fneur.2026.1955596 (PMC13710073; doi:10.3389/fneur.2026.1955596)
Supplement: Supplementary file 1 [file Data_Sheet_1.pdf]

Supplementary Material

1 SUPPLEMENTARY TABLES

Supplementary Table S1. CT Acquisition, Contrast Material, and Radiation Dose

Panel A. Scan and Reconstruction Parameters

Cohort and image role	Scanner	Scan mode	Tube voltage	Tube current–time product	Section thickness	Recon. kernel	Phase info.
Model development and primary evaluation cohorts: paired CTA	United Imaging Healthcare (UIH) uCT 960+	Helical	100 kVp	165–177 mAs	1.0 mm	H_SOFT_B	Single-phase CTA
Model development and primary evaluation cohorts: last-frame CTP input	UIH uCT 960+	Axial	100 kVp	69–74 mAs per frame	1.0 mm	H_VSOFT_B	21–22 frames; last frame ~57–62 s after first frame
Exploratory evaluation cohort: under-opacified CTA	Philips iCT 256 or Siemens SOMATOM Force	CTA	Siemens: 80 kVp; Philips: 100 kVp	Siemens: 82–184 mAs; Philips: 168–351 mAs	1.0 mm	Siemens: `Hr40s\3`, `Hv40s\3`; Philips: UB	Single-phase under-opacified CTA
Exploratory evaluation cohort: paired CTA	Philips iCT 256 or Siemens SOMATOM Force	CTA	120 kVp	Siemens: 252–580 mAs; Philips: 400 mAs	1.0 mm	Siemens: `Hr40s\3`, `Hv40s\3`; Philips: UB	Single-phase CTA

Panel B. Contrast Material Protocol

Cohort	Relationship between input and paired CTA
Model development and primary evaluation cohorts	CTP and CTA obtained in the same examination after a single contrast injection
Exploratory evaluation cohort	The under-opacified CTA and the paired CTA within 2 days derive from two separate contrast injections

Panel C. Radiation Dose

Examination	CTDIvol	Reference phantom
Model development and primary evaluation cohorts: paired CTA	9.2 mGy	32-cm body phantom
Model development and primary evaluation cohorts: complete CTP series	167.4 mGy	16-cm head phantom
Model development and primary evaluation cohorts: single CTP frame	˜7.6–8.0 mGy	16-cm head phantom
Exploratory evaluation cohort: under-opacified CTA	1.6–14.3 mGy	Not available
Exploratory evaluation cohort: paired CTA	36.1–82.9 mGy	Not available

Notes: CTDIvol, volume CT dose index; CTA, CT angiography; CTP, CT perfusion. CTDIvol values referenced to 16-cm and 32-cm phantoms are not directly comparable. The single-frame CTP value was estimated by dividing the complete-series CTDIvol by 21–22 frames.

Supplementary Table S2. Quantitative Image Quality in the Soft-Tissue Window (WW/WL 400/40)

Cohort	Model	n	SSIM	PSNR (dB)	nMSE
Primary evaluation cohort	RescueCTA	194	0.9160 ± 0.0132	30.78 ± 1.85	0.0241 ± 0.0179
Primary evaluation cohort	Pix2Pix	194	0.9245 ± 0.0159	31.70 ± 2.86	0.0224 ± 0.0220

Cohort	Model	n	SSIM	PSNR (dB)	nMSE
Primary evaluation cohort	Pix2PixHD	194	0.9109 ± 0.0177	32.32 ± 3.26	0.0219 ± 0.0298
Primary evaluation cohort	CycleGAN	194	0.9076 ± 0.0231	30.65 ± 3.88	0.0445 ± 0.1042
Exploratory evaluation cohort	RescueCTA	41	0.9218 ± 0.0325	28.85 ± 2.18	0.0374 ± 0.0110
Exploratory evaluation cohort	Pix2Pix	41	0.9330 ± 0.0299	29.63 ± 2.19	0.0315 ± 0.0095
Exploratory evaluation cohort	Pix2PixHD	41	0.9206 ± 0.0379	30.14 ± 3.78	0.0366 ± 0.0463
Exploratory evaluation cohort	CycleGAN	41	0.9214 ± 0.0311	31.58 ± 2.60	0.0208 ± 0.0102

Notes: Values are means ± standard deviations computed at the case level against the paired CTA. Higher SSIM and PSNR and lower nMSE indicate closer agreement with the paired CTA. CTA, CT angiography; CTP, CT perfusion; nMSE, normalized mean squared error; PSNR, peak signal-to-noise ratio; SSIM, structural similarity index measure; WW/WL, window width/window level.

Supplementary Table S3. Quantitative Image Quality Within Vessel Segmentation Masks

Model	SSIM ↑	PSNR (dB) ↑	nMSE ↓
RescueCTA	**0.8021 (0.7637–0.8401)**	**25.3729 (23.4670–27.4108)**	**2.4383 (1.6188–3.2839)**
Pix2PixHD	0.7536 (0.7079–0.8024)	22.8816 (18.6393–27.1340)	6.8666 (2.7471–11.6779)
CycleGAN	0.6543 (0.6119–0.7001)	12.9984 (12.1478–13.8722)	37.1532 (29.8210–45.1042)
Pix2Pix	0.6321 (0.5889–0.6802)	12.7568 (11.9281–13.6243)	39.3831 (31.3966–48.5988)

Notes: Values are means (bootstrap 95% CIs). ↑, higher is better; ↓, lower is better. Bold and underlined values denote the best and second-best means, respectively. CI, confidence interval; nMSE, normalized mean squared error; PSNR, peak signal-to-noise ratio; SSIM, structural similarity index measure.

Supplementary Table S4. Pairwise Comparisons of Subjective Image Quality Scores in the Primary Evaluation Cohort

Panel A. Overall Comparison Across the Five Image Conditions

Score domain	N	Friedman χ^2	df	p value
Overall image quality	194	575.01	4	<0.001
Artifact score	194	45.66	4	<0.001

Panel B. Prespecified Pairwise Comparisons for RescueCTA

Score domain	Comparison	RescueCTA score	Comparator score	RescueCTA higher/lower/tied	Wilcoxon W	Uncorrected p value	Holm-corrected p value
Overall image quality	RescueCTA vs paired CTA	3.90 ± 0.81	4.72 ± 0.57	12/132/50	1455.0	<0.001	<0.001
Overall image quality	RescueCTA vs Pix2Pix	3.90 ± 0.81	2.39 ± 0.80	170/1/23	56.5	<0.001	<0.001
Overall image quality	RescueCTA vs Pix2PixHD	3.90 ± 0.81	3.47 ± 0.92	85/11/98	1599.0	<0.001	<0.001
Overall image quality	RescueCTA vs CycleGAN	3.90 ± 0.81	1.87 ± 1.03	167/1/26	40.5	<0.001	<0.001
Artifact score	RescueCTA vs paired CTA	4.79 ± 0.66	4.96 ± 0.28	2/18/174	362.5	<0.001	<0.001
Artifact score	RescueCTA vs Pix2Pix	4.79 ± 0.66	4.71 ± 0.87	13/0/181	0.0	<0.001	<0.001
Artifact score	RescueCTA vs Pix2PixHD	4.79 ± 0.66	4.70 ± 0.91	13/2/179	368.0	0.004	0.004
Artifact score	RescueCTA vs CycleGAN	4.79 ± 0.66	4.66 ± 1.02	17/1/176	182.0	<0.001	<0.001

Notes: Scores were recorded on a paired 1–5 ordinal scale, and statistics are based on case-level consensus scores reached after discussion between the two readers. Overall differences were assessed using the Friedman test. Prespecified pairwise

comparisons between RescueCTA images and the other four image conditions used two-sided Wilcoxon signed-rank tests, with zero differences handled by the Pratt method, and asymptotic p values are reported. Holm correction was applied separately within each score domain. CTA, CT angiography; df, degrees of freedom; N, number of cases; W, Wilcoxon signed-rank statistic.

Supplementary Table S5. Interreader Agreement

Reading condition	Experience group	Binary agreement, n/N (%)	Binary κ	Laterality-adjusted agreement, n/N (%)	Laterality-adjusted κ
Last-frame CTP alone	3–5 years	168/194 (86.6%)	0.687	164/194 (84.5%)	0.683
Last-frame CTP alone	8–10 years	183/194 (94.3%)	0.855	181/194 (93.3%)	0.844
Last-frame CTP + RescueCTA	3–5 years	185/194 (95.4%)	0.897	180/194 (92.8%)	0.859
Last-frame CTP + RescueCTA	8–10 years	184/194 (94.8%)	0.882	183/194 (94.3%)	0.883
Paired CTA	3–5 years	188/194 (96.9%)	0.930	188/194 (96.9%)	0.938
Paired CTA	8–10 years	190/194 (97.9%)	0.953	189/194 (97.4%)	0.947

Notes: Binary agreement was computed using the clinical-failure definition given in the Methods. Laterality-adjusted agreement additionally required agreement on the side of occlusion. CTA, CT angiography; CTP, CT perfusion; κ denotes Cohen kappa.

Supplementary Table S6. Diagnostic Results in the Exploratory Evaluation Cohort

Reading condition	Reader	No. of readings	TP	FN	TN	FP	AUC	Sens., n/N (%)	Spec., n/N (%)	Acc., n/N (%)	Non-interp., n
Under-opacified CTA alone	Reader 3	41	1	1	35	4	0.833	1/2 (50.0%)	35/39 (89.7%)	36/41 (87.8%)	0
Under-opacified CTA alone	Reader 4	41	2	0	33	6	0.949	2/2 (100.0%)	33/39 (84.6%)	35/41 (85.4%)	0

Reading condition	Reader	No. of readings	TP	FN	TN	FP	AUC	Sens., n/N (%)	Spec., n/N (%)	Acc., n/N (%)	Non-interp., n
Under-opacified CTA alone	Pooled	82	3	1	68	10	0.889	3/4 (75.0%)	68/78 (87.2%)	71/82 (86.6%)	0
Under-opacified CTA + RescueCTA	Reader 3	41	2	0	37	2	0.987	2/2 (100.0%)	37/39 (94.9%)	39/41 (95.1%)	0
Under-opacified CTA + RescueCTA	Reader 4	41	2	0	36	3	0.974	2/2 (100.0%)	36/39 (92.3%)	38/41 (92.7%)	0
Under-opacified CTA + RescueCTA	Pooled	82	4	0	73	5	0.981	4/4 (100.0%)	73/78 (93.6%)	77/82 (93.9%)	0
Paired CTA	Reader 3	41	2	0	39	0	1.000	2/2 (100.0%)	39/39 (100.0%)	41/41 (100.0%)	0
Paired CTA	Reader 4	41	2	0	39	0	1.000	2/2 (100.0%)	39/39 (100.0%)	41/41 (100.0%)	0
Paired CTA	Pooled	82	4	0	78	0	1.000	4/4 (100.0%)	78/78 (100.0%)	82/82 (100.0%)	0

Notes: Descriptive analysis only. Readers 3 and 4 have 8 to 10 years of experience. AUC, area under the receiver operating characteristic curve; CTA, CT angiography; CTP, CT perfusion; FN, false negative; FP, false positive; TN, true negative; TP, true positive.

Supplementary Table S7. Reader-Level Diagnostic Performance for Target LVO Diagnosis in the Primary Evaluation Cohort

Reader	Experience	AUC	Strict AUC	TP/FN/TN/FP	Sens., n/N (%)	Spec., n/N (%)	Acc., n/N (%)	Balanced accuracy	Non-interp., n/N (%)
**Last-frame CTP alone**									
Reader 1	3–5 y	0.815	0.709	33/28/111/22	33/61 (54.1%)	111/133 (83.5%)	144/194 (74.2%)	68.8%	13/194 (6.7%)
Reader 2	3–5 y	0.788	0.660	31/30/107/26	31/61 (50.8%)	107/133 (80.5%)	138/194 (71.1%)	65.6%	11/194 (5.7%)
Reader 3	8–10 y	0.903	0.838	38/23/122/11	38/61 (62.3%)	122/133 (91.7%)	160/194 (82.5%)	77.0%	8/194 (4.1%)
Reader 4	8–10 y	0.893	0.816	38/23/119/14	38/61 (62.3%)	119/133 (89.5%)	157/194 (80.9%)	75.9%	7/194 (3.6%)
**Last-frame CTP + RescueCTA**									
Reader 1	3–5 y	0.900	0.831	46/15/115/18	46/61 (75.4%)	115/133 (86.5%)	161/194 (83.0%)	80.9%	8/194 (4.1%)
Reader 2	3–5 y	0.929	0.853	45/16/114/19	45/61 (73.8%)	114/133 (85.7%)	159/194 (82.0%)	79.7%	8/194 (4.1%)
Reader 3	8–10 y	0.928	0.900	52/9/123/10	52/61 (85.2%)	123/133 (92.5%)	175/194 (90.2%)	88.9%	4/194 (2.1%)
Reader 4	8–10 y	0.973	0.935	54/7/123/10	54/61 (88.5%)	123/133 (92.5%)	177/194 (91.2%)	90.5%	4/194 (2.1%)
**Paired CTA**									
Reader 1	3–5 y	0.930	0.905	51/10/121/12	51/61 (83.6%)	121/133 (91.0%)	172/194 (88.7%)	87.3%	1/194 (0.5%)
Reader 2	3–5 y	0.944	0.927	52/9/123/10	52/61 (85.2%)	123/133 (92.5%)	175/194 (90.2%)	88.9%	1/194 (0.5%)
Reader 3	8–10 y	0.940	0.940	57/4/127/6	57/61 (93.4%)	127/133 (95.5%)	184/194 (94.8%)	94.5%	0/194 (0.0%)
Reader 4	8–10 y	0.977	0.968	58/3/129/4	58/61 (95.1%)	129/133 (97.0%)	187/194 (96.4%)	96.0%	0/194 (0.0%)

Notes: Readers 1 and 2 have 3 to 5 years of experience; readers 3 and 4 have 8 to 10 years of experience. Binary metrics use the clinical-failure definition: scores of 4–5 were positive, scores of 1–2 were negative, and non-interpretable readings and scores of 3 were counted as errors according to the reference-standard direction. A true positive required both a positive judgment and correct identification of the side of occlusion; positive judgments with the wrong side were counted as false negatives. AUC excludes non-interpretable readings. Strict AUC penalizes non-interpretable and indeterminate readings and positive judgments identifying the wrong side of occlusion. AUC, area under the receiver operating characteristic curve; CTA, CT angiography; CTP, CT perfusion; FN, false negative; FP, false positive; LVO, large-vessel occlusion; TN, true negative; TP, true positive.

Supplementary Table S8. Component Ablation Analysis of RescueCTA Using the Whole-Field Vascular-Window Protocol

Configuration	HRNet generator	Multi-scale PatchGAN	VGG/FM losses	Progressive schedule	SSIM	PSNR (dB)	nMSE
Full RescueCTA	✓	✓	✓	✓	0.9150 ± 0.0132	30.78 ± 1.87	0.0265 ± 0.0206
w/o HRNet generator	×	✓	✓	✓	0.8066 ± 0.0134	30.44 ± 0.46	0.0168 ± 0.0019
w/o multi-scale PatchGAN	✓	×	✓	✓	0.8089 ± 0.0145	30.62 ± 0.46	0.0160 ± 0.0020
w/o VGG/FM losses	✓	✓	×	✓	0.4529 ± 0.0195	9.36 ± 0.12	2.0663 ± 0.0798
w/o progressive schedule	✓	✓	✓	×	0.4529 ± 0.0195	9.36 ± 0.12	2.0663 ± 0.0798

Notes: Values are case-level macro means ± standard deviations using the whole-field vascular window (WW/WL 400/50). Higher SSIM and PSNR and lower nMSE indicate closer agreement with the paired CTA. The Full RescueCTA value is the original-paper reference result; the component variants were obtained under the component-ablation training protocol. ✓ indicates that the component was retained and × indicates that it was removed or replaced. The w/o HRNet and w/o multi-scale PatchGAN variants used a U-Net generator and a single-scale PatchGAN, respectively. CTA, CT angiography; FM, feature matching; nMSE, normalized mean squared error; PSNR, peak signal-to-noise ratio; SSIM, structural similarity index measure; WW/WL, window width/window level.

2 SUPPLEMENTARY FIGURES

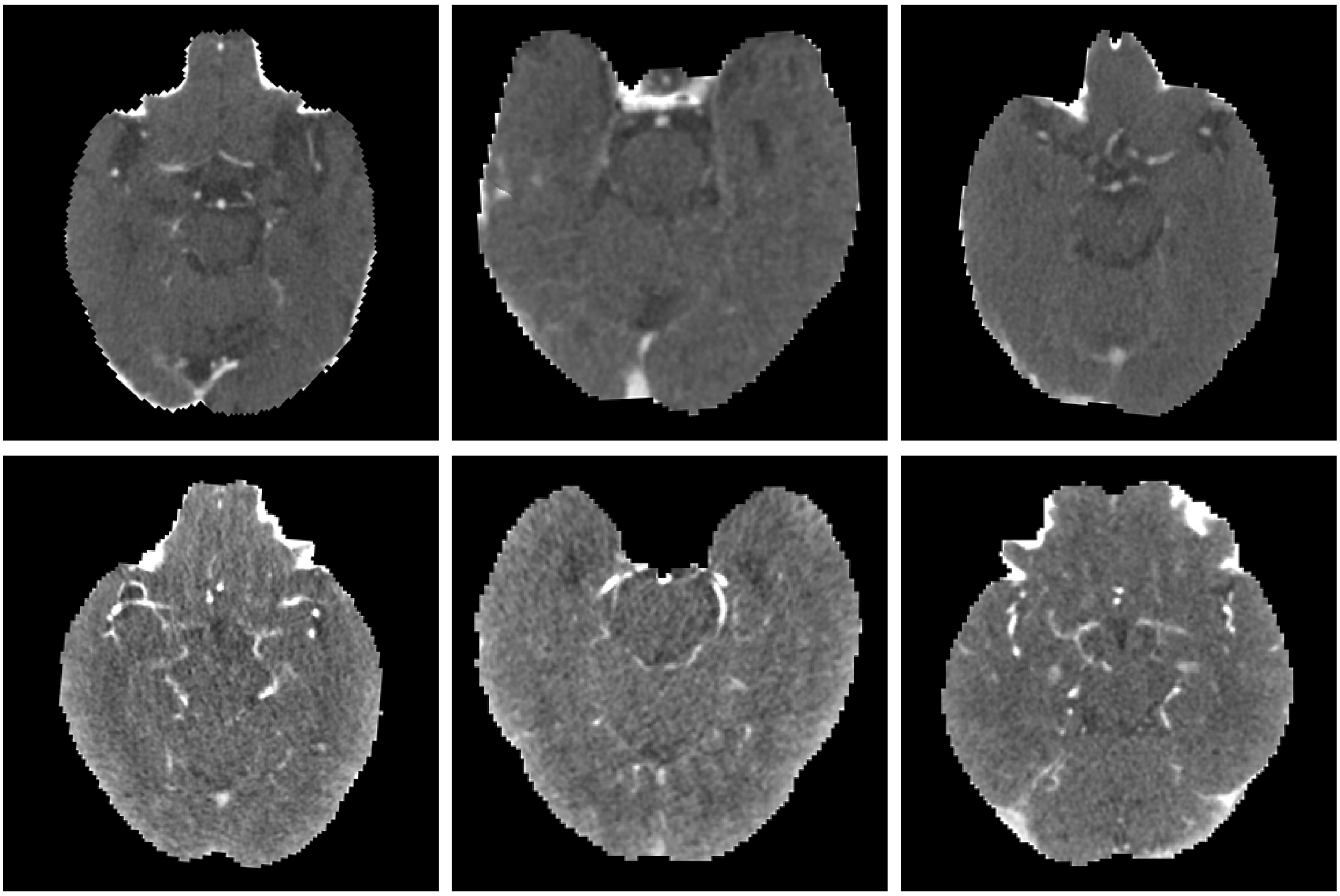

Figure S1. Arterial Enhancement of the Source Images in the Two Evaluation Cohorts. Representative comparison between the simulated last-frame CTP inputs in the primary evaluation cohort and the truly under-opacified CTA inputs in the exploratory evaluation cohort. The top row shows 3 last-frame CTP input cases from the primary evaluation cohort, and the bottom row shows 3 truly under-opacified CTA input cases from the exploratory evaluation cohort. All images are displayed at the same window width/window level. CTA, CT angiography; CTP, CT perfusion.

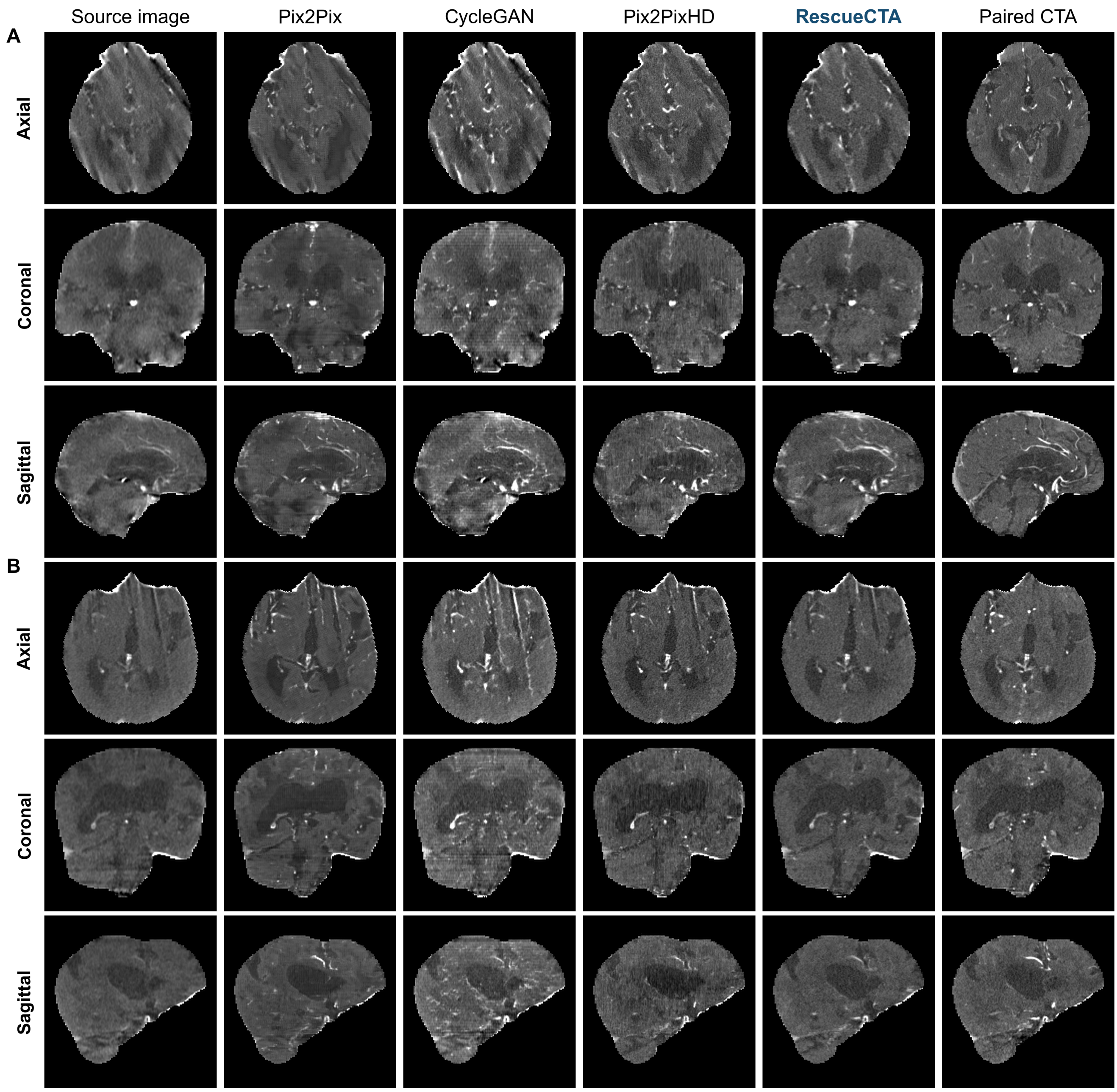

Figure S2. Two Representative Cases With Conspicuous Artifacts. Two representative cases with conspicuous artifacts. Axial, coronal, and sagittal images are shown for each case. All images are displayed at the same window width/window level.

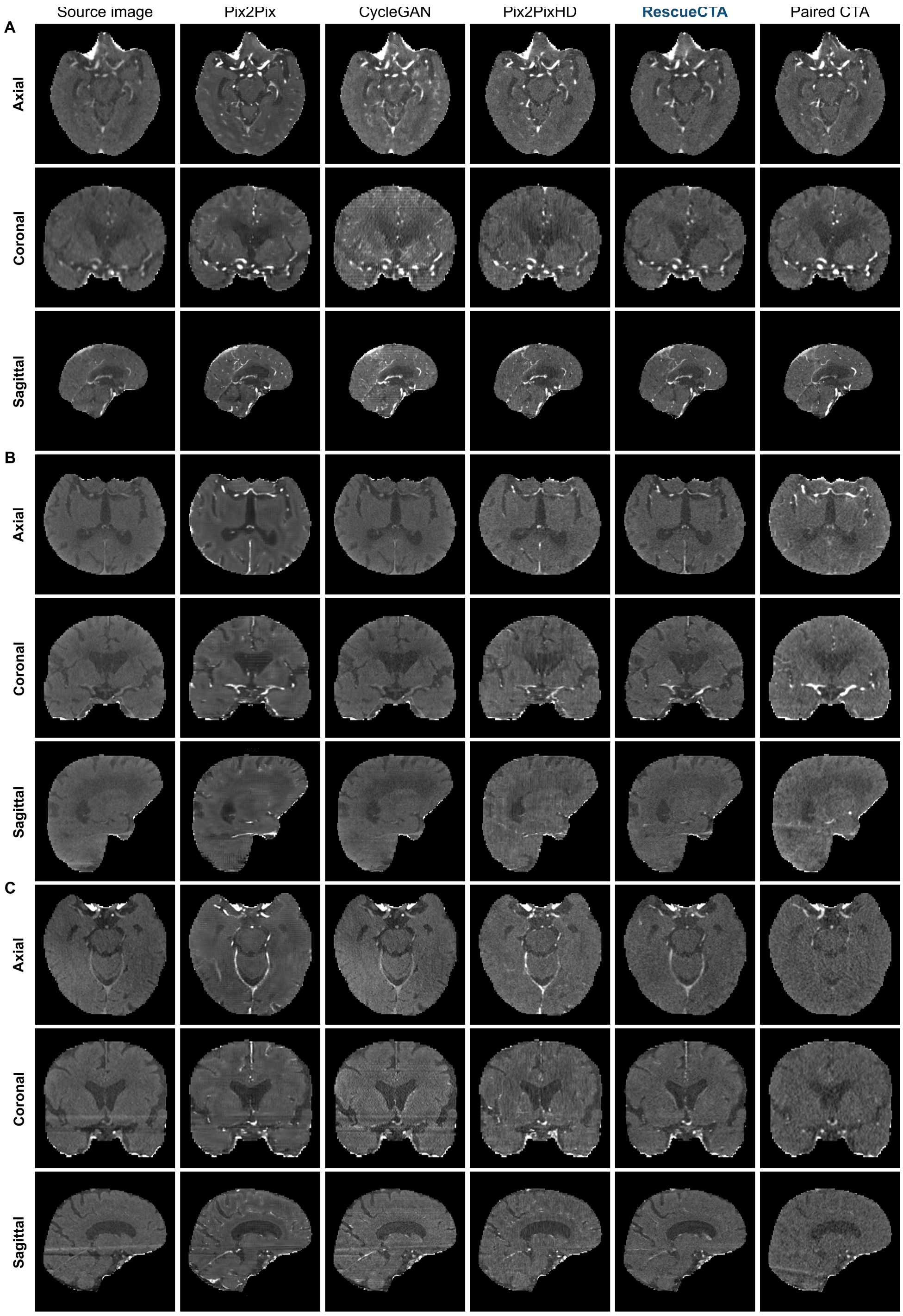

Figure S3. Three Representative Cases Without Conspicuous Artifacts. Axial, coronal, and sagittal images are shown for each case. All images are displayed at the same window width/window level.

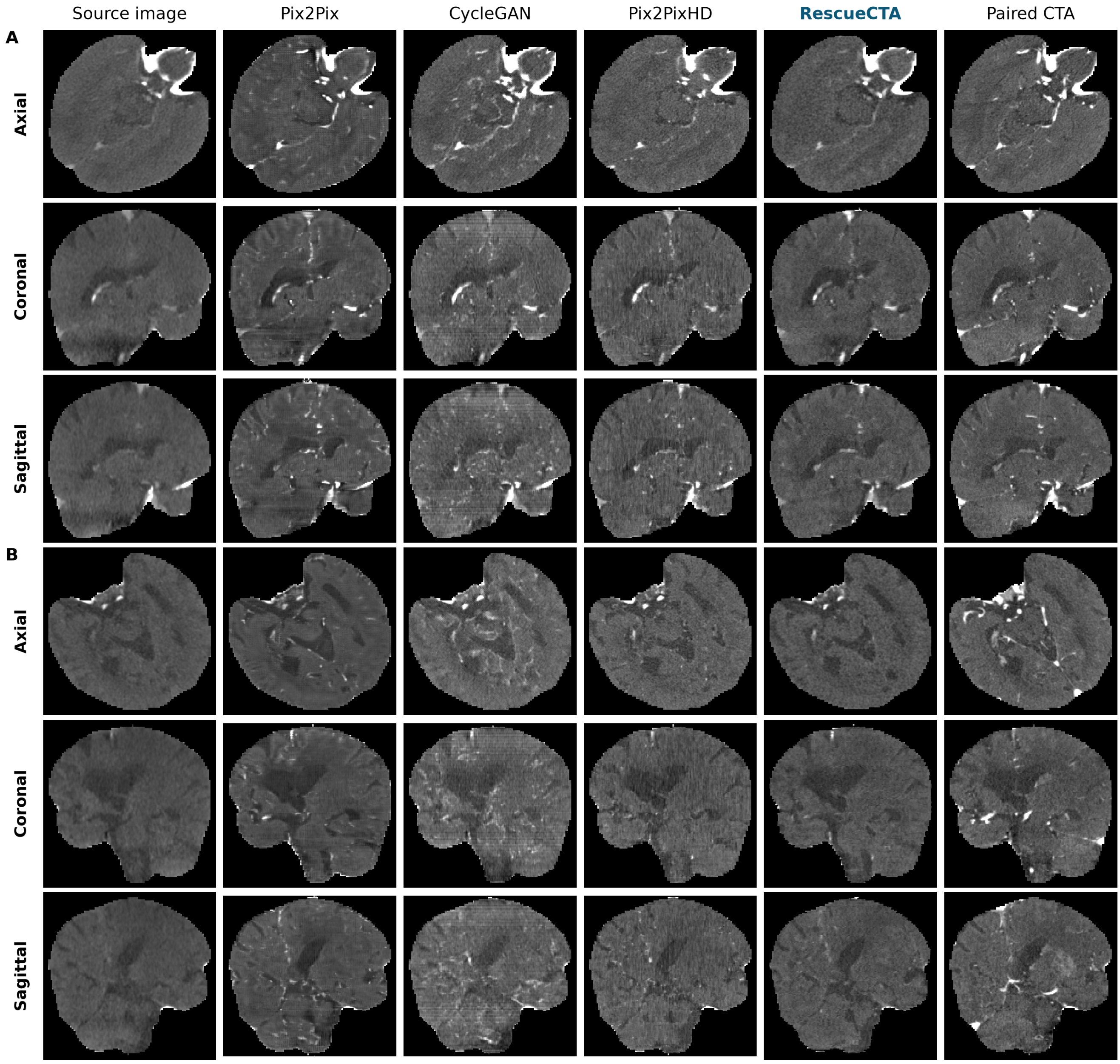

Figure S4. Representative Failure Cases. (A, B) Two representative generation-failure cases. In both cases, some vessels visible on paired CTA were not fully reconstructed on RescueCTA. For each case, axial, coronal, and sagittal views are shown for the source image, Pix2Pix, CycleGAN, Pix2PixHD, RescueCTA, and paired CTA. All images are displayed at the same window width/window level.

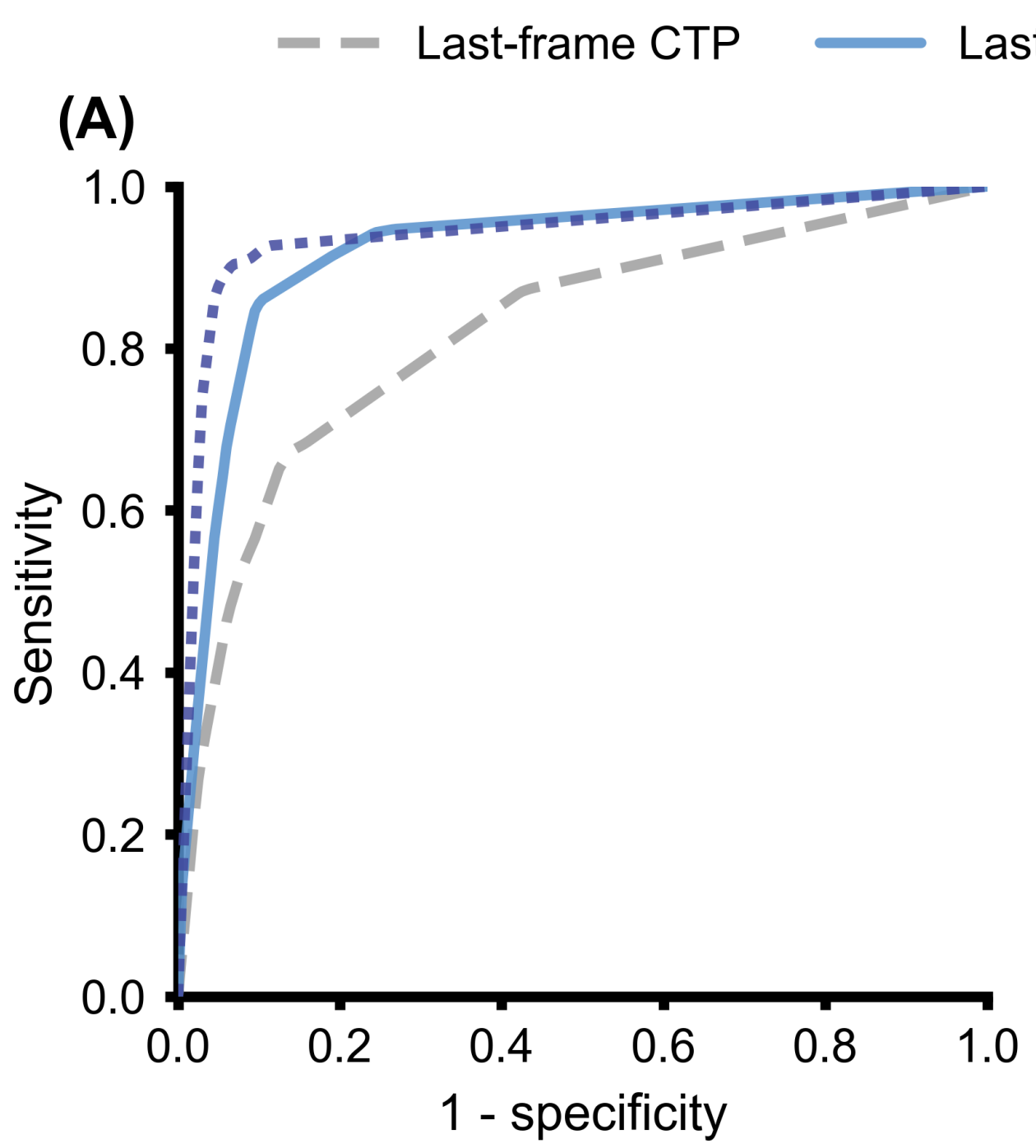

Figure S5. Component Sensitivity Analyses of the ROC. (A) Failure-penalized ROC sensitivity analysis; non-interpretable and indeterminate readings were assigned an erroneous outcome according to the direction of the reference standard, without penalization of wrong-side judgments. (B) Correct-side ROC sensitivity analysis; in reference-standard-positive cases, a positive judgment with the wrong side was counted as an error, whereas non-interpretable readings were excluded. Gray, blue, and indigo curves denote reading last-frame CTP images alone, last-frame CTP images + RescueCTA, and the paired CTA, respectively. CTA, CT angiography; CTP, CT perfusion; ROC, receiver operating characteristic.

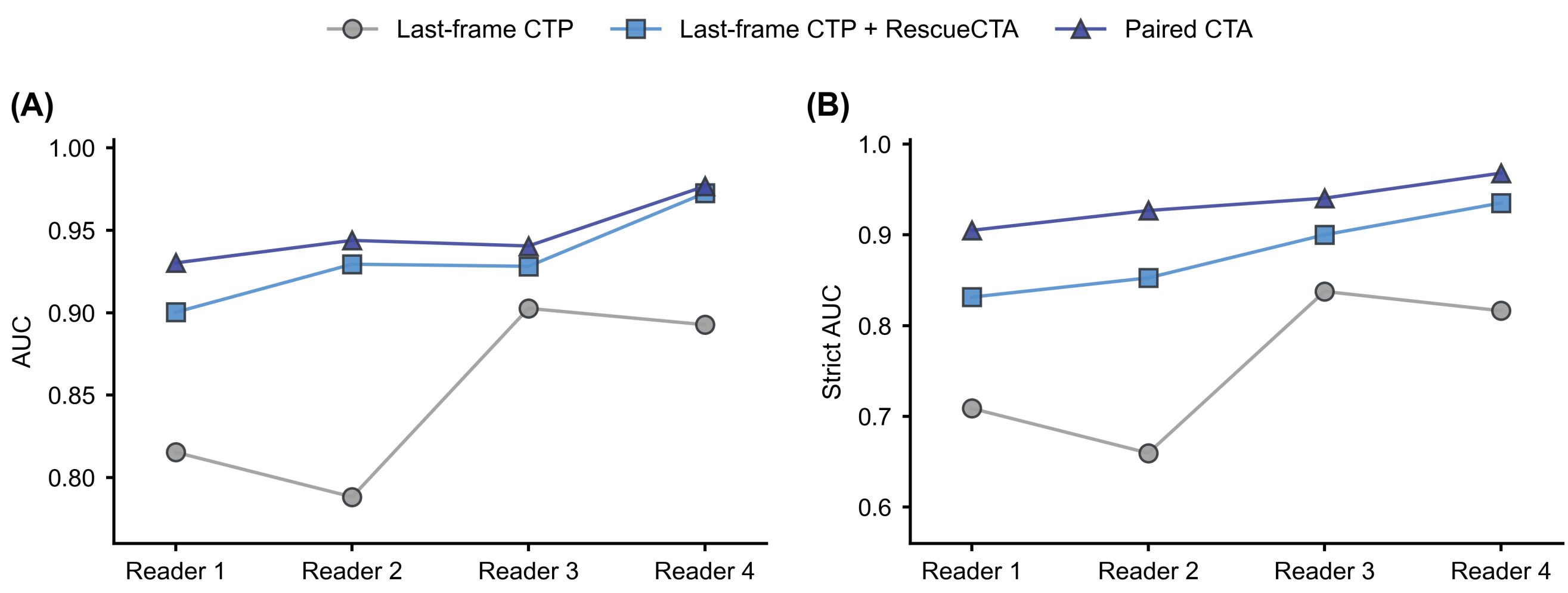

Figure S6. Reader-Level AUC and Strict AUC in the Primary Evaluation Cohort. AUC (A) and strict AUC (B) for each of the 4 readers under the conditions of reading last-frame CTP images alone (gray), combined reading of last-frame CTP images plus RescueCTA images (blue), and the paired CTA (indigo). Readers 1 and 2 have 3 to 5 years of experience, and readers 3 and 4 have 8 to 10 years of experience. Note the different y-axis ranges in the two panels. AUC, area under the receiver operating characteristic curve; CTA, CT angiography; CTP, CT perfusion.

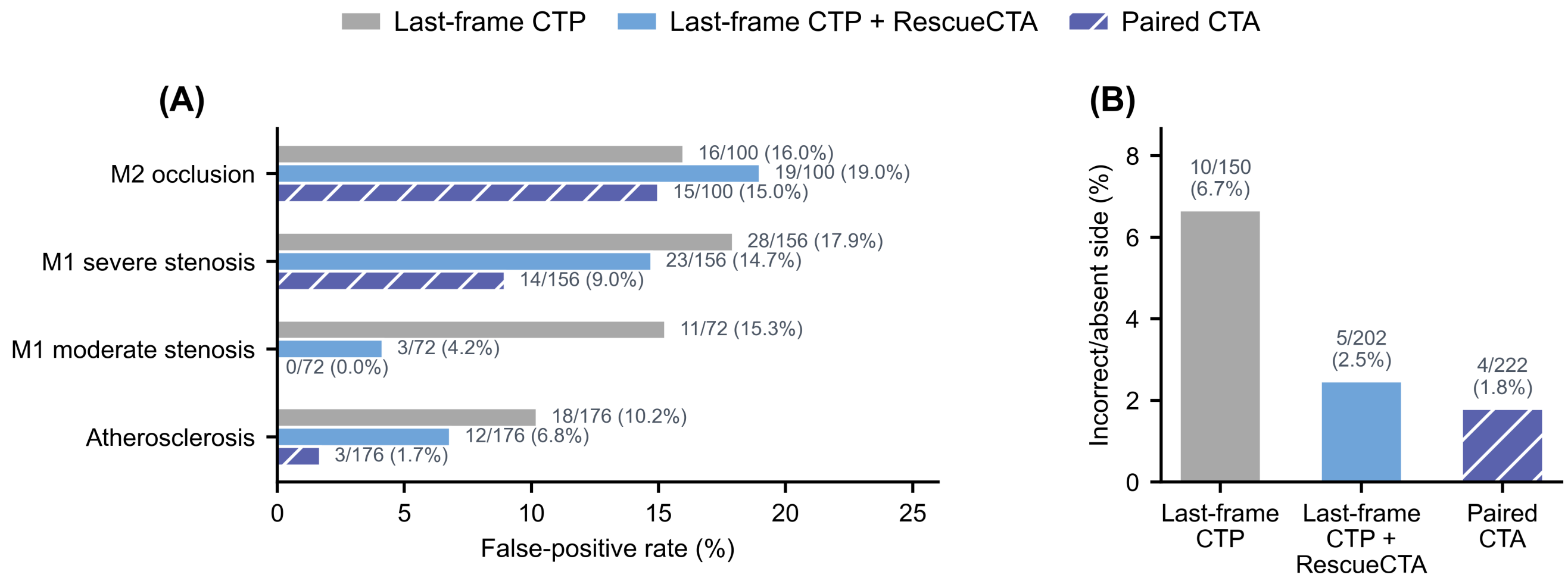

Figure S7. Error Patterns in the Primary Evaluation Cohort. (A) Principal sources of clinical-failure false positives among reference-standard-negative reader–case records. Bar labels are n/N (%): the numerator is the number of records in that lesion category counted as false positives under the clinical-failure definition, including positive judgments and non-interpretable or indeterminate readings, and the denominator is the total number of reader–case records in that lesion category. Cases with mild M1 stenosis and normal vessels produced no false-positive records under any of the three reading conditions. (B) Proportion of positive reader–case records with incorrect laterality among reference-standard-positive records judged positive. Bar labels are n/N (%): the numerator is the number of positive judgments with incorrect laterality, and the denominator is the total number of target-LVO reader–case records judged positive under that reading condition. Gray, blue, and indigo denote reading last-frame CTP images alone, last-frame CTP images + RescueCTA, and the paired CTA, respectively. CTA, CT angiography; CTP, CT perfusion; LVO, large-vessel occlusion.
